# Identification of a metabolic reprogramming‐related signature associated with prognosis and immune microenvironment of head and neck squamous cell carcinoma by in silico analysis

**DOI:** 10.1002/cam4.4670

**Published:** 2022-03-18

**Authors:** Weijie Qiang, Yifei Dai, Xiaoyan Xing, Xiaobo Sun

**Affiliations:** ^1^ Institute of Medicinal Plant Development Chinese Academy of Medical Sciences and Peking Union Medical College Beijing China; ^2^ Key Laboratory of New Drug Discovery Based on Classic Chinese Medicine Prescription Chinese Academy of Medical Sciences Beijing China; ^3^ School of Medicine Tsinghua University Beijing China

**Keywords:** head and neck squamous cell carcinoma, metabolic reprogramming, metabolism‐related genes, prognostic signature, tumor immune microenvironment

## Abstract

**Background:**

Metabolic reprogramming is one of the essential features of tumorigenesis. Herein, this study aimed to develop a novel metabolism‐related gene signature for head and neck squamous cell carcinoma (HNSCC) patients.

**Methods:**

The transcriptomic and clinical data of HNSCC samples were collected from The Cancer Genome Atlas (TCGA) and GSE65858 datasets. The metabolism‐related gene‐based prognostic signature (MRGPS) was constructed by the Least Absolute Shrinkage and Selection Operator (LASSO) regression model. The time‐dependent receiver operating characteristic (ROC) and Kaplan‐Meier (K‐M) survival curves were plotted for evaluating its predicting performance. At the same time, univariate along with multivariate analysis was carried out to explore its correlation with clinicopathologic factors. Furthermore, GSEA analysis was performed to explore the signaling pathways affected by MRGPS. We also analyzed the associations of MRGPS with the tumor immune microenvironment (TIME), as well as identified potential compounds via Connectivity Map (CMap) and molecular docking.

**Results:**

A total of 12 differentially expressed metabolism‐related genes were identified and selected to construct the MRGPS. Notably, this signature performed well in predicting HNSCC patients’ survival and could serve as an independent prognostic factor in multiple datasets. In addition to the metabolism‐related pathway, this signature could also affect some immune‐related pathways. The results indicated that MRGPS is correlated with immune cells infiltration and anti‐cancer immune response. Furthermore, we identified cephaeline as a potential therapeutic compound for HNSCC.

**Conclusion:**

Taken together, we established an MRGs‐based signature that has the potential to predict the clinical outcome and immune microenvironment, which help to search for potential combination immunotherapy compounds and provide a promising therapeutic strategy for treating HNSCC patients.

## Graphical abstract

In this research, by comprehensively analyzing transcriptomic and clinical data of HNSC samples, a metabolism‐related gene‐based prognostic signature (MRGPS) was established and sufficiently validated in multiple datasets. Also, this study explored the association of MRGPS with multiple clinicopathologic factors and constructed a prognostic nomogram. Interestingly, our GSEA analysis found that MRGPS was closely related to the immune‐related pathways. Furthermore, we explored the relationships between MRGPS and tumor immune microenvironment (TIME), as well as immunotherapy response.

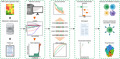

## 1 INTRODUCTION

Head and neck squamous cell carcinoma (HNSCC) represents a frequently occurring malignant tumor globally. Due to its complex and diverse anatomical parts, involving appearance and various essential physiological functions, it seriously affects patients’ life quality. According to the WHO statistics, there are 830,000 new cases along with 430,000 deaths across the world in 2018.^1^ Furthermore, more than 70% of HNSCC patients have already been at the moderate and advanced stages when they are diagnosed, with a low 5‐year survival rate of <40%.^2^ Thus, it is urgently needed to develop a novel predicting approach in order to enhance the prognostic outcome of HNSCC patients.

Metabolic reprogramming is extensively regarded as a hallmark of cancer, which contributes to tumorigenesis in HNSCC.^3^ Numerous studies have demonstrated that it is possible to use metabolic phenotypes for imaging tumors, providing prognosis data, and treating tumors.^4^ Targeting specific metabolic pathways can be an effective cancer treatment strategy. For example, both 5‐fluorouracil (5‐FU, pyrimidine analog) and cytarabine (antimetabolite nucleoside analog) exhibit favorable antitumor effects.^5^, ^6^ Noteworthily, it has been previously revealed that HNSCC progression is closely related to several metabolic pathways, and energy metabolism may become a promising therapeutic target for HNSCC patients.^3^, ^7^ Nonetheless, for metabolism‐related genes (MRGs) that mediate metabolic reprogramming, their expression patterns and clinical values in HNSCC remain unclear. Therefore, systematically analyzing the characteristics and clinical significance of MRGs may be crucial for treating HNSCC.

**FIGURE 1:**
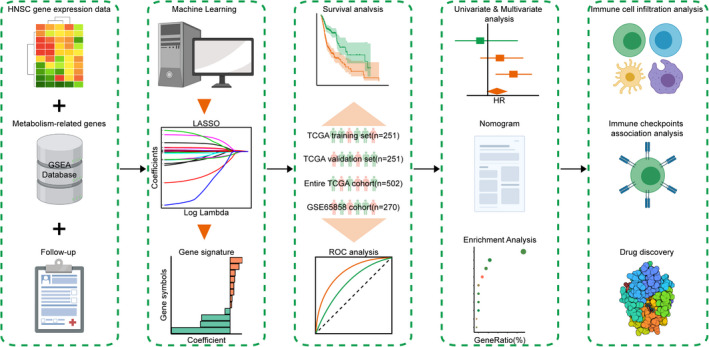
Experimental technical roadmap. The whole process is divided into five steps: data collection, signature construction, verification of predictive potential, correlation analysis, and identification of potential compounds

In this study, by comprehensively analyzing the transcriptomic and clinical data of HNSCC samples, a metabolism‐related gene‐based prognostic signature (MRGPS) was formed and sufficiently validated in a variety of data sets. Afterward, this study explored the association of MRGPS with multiple clinicopathologic factors and constructed a prognostic nomogram. Interestingly, our gene set enrichment analysis (GSEA) concluded that MRGPS was tightly associated with the immune‐related pathways. Hence, we explored the relationships between MRGPS and tumor immune microenvironment (TIME) as well as anticancer immune response. Besides, we identified the potential compound specific to MRGPS. Figure 1 summarizes the experimental technical roadmap.

## 2 METHODS

### 2.1 Collection of HNSCC sample information and MRGs

The Cancer Genome Atlas (TCGA) cohort (*n* = 502) from TCGA data portal and the GSE65858 cohort (*n* = 270) from Gene Expression Omnibus (GEO) were selected and used for collecting transcriptomic and clinical data of HNSCC samples.^8^, ^9^ Thereafter, at a random order, the entire TCGA cohort was subdivided into the TCGA training set (*n* = 251) and TCGA validation set (*n* = 251). The entire TCGA cohort and GSE65858 cohort were also applied as the internal testing set and external testing set, respectively. In the TCGA cohort, there were 413 human papillomaviruses (HPV) negative cases and 70 HPV‐positive cases, with 19 cases of unlabeled HPV status. Among the GSE65858 cohort, there were 196 HPV‐negative cases and 73 HPV‐positive cases, with 1 case of unlabeled HPV status. Table Figure S1 displayed patient demographics and clinical features of the included data sets. Furthermore, 1454 specifical MRGs involved in 69 metabolism‐related pathways were obtained from c2.cp.kegg.v7.2.symbols.gmt at the GSEA website.^10^

### 2.2 Identification of DEMRGs

Using the thresholds of |log2(Fold Change)| >1 and *p* < 0.05, differentially expressed genes (DEGs) were identified between 502 HNSCC samples and 44 noncarcinoma samples with the use of R package “limma”.^11^ Moreover, the differentially expressed metabolism‐related genes (DEMRGs) were subsequently extracted from all DEGs. The volcano plot and Venn diagram of DEMRGs were drawn with the use of the R package “ggplot2”^12^ and an online tool (http://bioinformatics.psb.ugent.be/webtools/Venn/), respectively.

### 2.3 Construction of MRGs‐based prognostic signature (MRGPS) and functional enrichment analysis

The associations of DEMRGs with overall survival (OS) were analyzed using the univariate Cox proportional hazard regression model in the TCGA training set, followed by the identification of prognosis‐related DEMRGs with the cutoff of *p* < 0.05. Thereafter, the most optimal gene set was selected using the Least Absolute Shrinkage and Selection Operator (LASSO) penalized Cox proportional hazards regression through R package “glmnet.” Thereinto, the optimal gene set was used to construct the MRGPS, and the MRGs incorporated into the MRGPS were regarded as hub MRGs. In addition, differential expression of these hub MRGs was also confirmed through the Oncomine database.^13^ Ultimately, the risk score of each HNSCC patient was determined by the following: risk score = [Gene 1 expression level × coefficient] + [Gene 2 expression level × coefficient] + … + [Gene *n* expression level × coefficient]. Later, all HNSCC patients were categorized as the low‐ or high‐risk group on the basis of the median risk score.

Besides, this study carried out the functional enrichment analysis with the purpose of investigating the biological roles of these hub MRGs, including Gene Ontology (GO) and Kyoto Encyclopedia of Genes and Genomes (KEGG) through the Database for Annotation, Visualization, and Integrated Discovery (DAVID) 6.8. Therefore, the GO terms consist of biological process (BP), molecular function (MF), and cellular component (CC). Using *p* < 0.05 as the screening criterion, the R package “ggplot2” was adopted for enriching and visualizing the GO terms and KEGG pathways.

### 2.4 Evaluation of MRGPS‐predictive capability

In order to evaluate the predictive capability of MRGPS, the Kaplan–Meier (K–M) survival curves of the low‐risk and high‐risk groups were plotted by R package “survival”.^14^ In addition, the time‐dependent receiver‐operating characteristic (ROC) curves (containing 1‐, 3‐, and 5‐year survival) were plotted via R package “survival ROC,” aiming to determine the specificity and sensitivity of MRGPS.^15^

### 2.5 Associations between MRGPS and clinicopathologic variables

In the entire TCGA and GSE65858 cohorts, R package “survival” was utilized for univariate and multivariate analyses of MRGPS and clinicopathologic variables on OS.^14^ Besides, the relationships between MRGPS and clinicopathological variables were evaluated by independent *t*‐tests. Combining these clinicopathological variables with MRGPS, this study attempted to construct a prognostic nomogram via R package “rms,” aiming to quantitatively assess individual HNSCC patients’ survival probability.^16^

### 2.6 Gene set enrichment analysis

GSEA has been developed as the calculation approach to determining the significant and consistent differences in a previously defined gene set between two distinct biological states (such as phenotypes). With the cutoff of FDR <0.25, GSEA analysis was performed to elucidate the enriched BPs, CCs, and MFs, together with the related KEGG pathways in the low‐ or high‐risk group.

### 2.7 Analysis of immune cell infiltration and immunotherapy efficacy

To analyze the association between MRGPS and immune cell infiltration, we adopted CIBERSORT to estimate the infiltration levels of 22 immune cell subtypes in the low‐ and high‐risk groups.^17^ Meanwhile, the correlations of hub MRGs with immune cell infiltration were analyzed. In addition, the survival analysis was conducted to explore the relationship between the infiltration levels of 22 immune cell subtypes and the prognosis of HNSCC patients. Immunotherapy is a promising treatment strategy for HNSCC patients, which can be conceptualized as seven‐step anticancer immune responses via the TIP database (http://biocc.hrbmu.edu.cn/TIP/).^18^ Therefore, we performed a correlation analysis between anticancer immune responses and MRGPS. Moreover, immune checkpoint inhibitor (ICI) is an essential component of immunotherapy, so we explored the differences in expressing several prominent immune checkpoints in both groups.

### 2.8 Connectivity map analysis

To identify the candidate compounds that target MRGPS, connectivity map (CMap) (https://portals.broadinstitute.org/cmap/), an online tool used to predict the compounds that activate or inhibit a previously defined gene signature in diverse cell lines, was adopted in this study.^19^ With the pattern‐matching algorithms, a positive score represents the promoting effect of the compounds on the query signature, whereas a negative score indicates the inhibiting effect. Furthermore, the mechanism of actions (MoAs) information of these compounds was collected from the CMap tool “repurposing” (https://clue.io/repurposing‐app) in order to explore their shared MoAs.

### 2.9 Construction of the PPI network and molecular docking

With the purpose of screening the key target, all MRGPS‐related genes were mapped to the STRING database (https://string‐db.org/) to construct a protein–protein interaction (PPI) network,^20^ followed by the topological analysis based on the Network Analyzer plug‐in contained in Cytoscape. Besides, molecular docking was carried out to screen candidate compounds. Schrodinger’s Glide module was applied for molecular docking, and MM‐GBSA analysis was applied to calculate the relative binding free energy of candidate compounds with the key target.^21^

### 2.10 Statistical analysis

The R package “survival” was used for univariate and multivariate Cox regression analyses.^14^ The hazard ratios together with the corresponding 95% confidence intervals were collected. In addition, an independent *t* test was adopted for comparing the heterogeneities among diverse clinical factors. A difference of *p* < 0.05 was regarded to present statistical significance.

## 3 RESULTS

### 3.1 Construction of MRGPS

**FIGURE 2:**
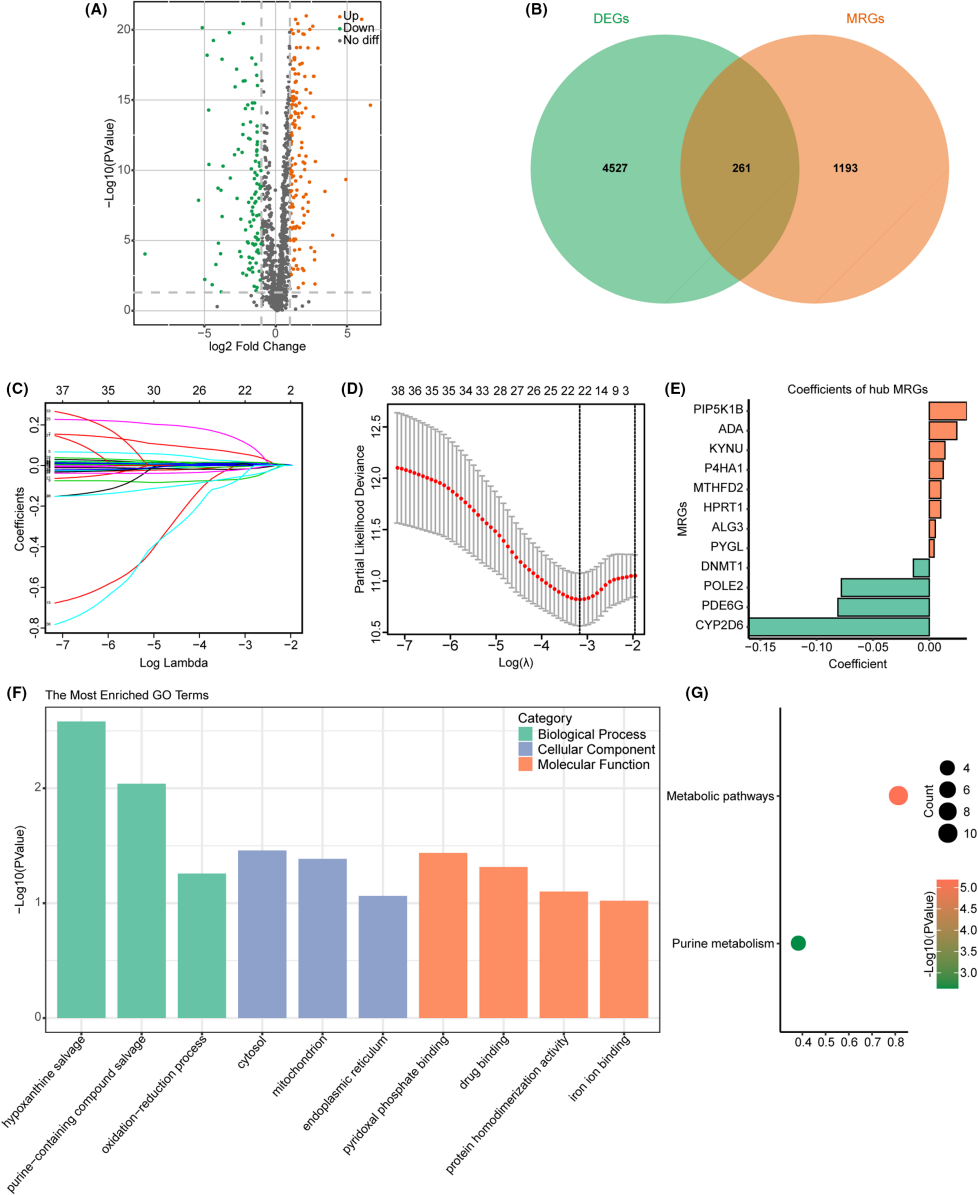
Establishment of MRGPS and enrichment analysis of hub MRGs. (A) Volcano plot regarding the DEMRGs in HNSCC samples versus normal samples. (B) Venn diagram visualizing the intersections of DEGs with MRGs. (C) LASSO coefficient profiles in LASSO Cox regression analysis. (D) Screening of the tuning parameter (lambda) from the LASSO model through 10‐fold cross‐validation according to the minimal OS threshold. (E) Established coefficient of hub MRGs used for MRGPS. (F) GO enrichment analysis for the 12 identified hub MRGs. The green, blue, and orange modules stand for BP, CC, and MF terms, separately. (G) KEGG enrichment analysis of 12 hub MRGs. BP, biological process; CC, cellular component; DEGs, differentially expressed genes; DEMRGs, differentially expressed metabolism‐related genes; GO, Gene Ontology; HNSCC, head and neck squamous cell carcinoma; LASSO, Least Absolute Shrinkage and Selection Operator; MF, molecular function; MRGPS, metabolism‐related gene‐based prognostic signature; MRGs, metabolism‐related genes; OS, overall survival

By comparing HNSCC samples and normal samples, we obtained 4788 DEGs. Meanwhile, we acquired 1454 MRGs via the GSEA website. Ultimately, altogether 261 DEMRGs were extracted, among which, 146 showed upregulation, whereas 115 presented downregulation (Figure 2A,B).

There were 38 DEMRGs in the TCGA training set significantly associated with OS of HNSCC cases (*p* < 0.05, Table S2). The most appropriate tuning parameter (lambda) from the LASSO model was chosen to prevent overfitting (Figure 2C,D), which contributed to the production of the optimal model. In total, 12 genes were selected to as hub MRGs, including *PIP5K1B* (Phosphatidylinositol‐4‐phosphate 5‐kinase type‐1 beta), *ADA* (adenosine deaminase), *KYNU* (Kynureninase), *P4HA1* (Prolyl 4‐hydroxylase subunit alpha‐1), *MTHFD2* (Methylenetetrahydrofolate dehydrogenase 2), *HPRT1* (hypoxanthine phosphoribosyltransferase 1), *ALG3* (alpha‐1,3‐ mannosyltransferase), *PYGL* (Glycogen phosphorylase L), *DNMT1* (DNA methyltransferase 1), *POLE2* (DNA polymerase epsilon 2), *PDE6G* (phosphodiesterase 6G), and *CYP2D6* (cytochrome P450 family 2 subfamily D member 6) (Figure 2E). Their expression was markedly upregulated in diverse tumor tissues compared with that in noncarcinoma tissues (Figure S1). Afterward, the MRGPS was established based on these 12 hub MRGs expression profiles and their Cox regression coefficients as follows: risk score = [*PIP5K1B* expression level × 0.033363] + [*ADA* expression level × 0.024469] + [*KYNU* expression level × 0.013974] + [*P4HA1* expression level × 0.012453] + [*MTHFD2* expression level × 0.010309] + [*HPRT1* expression level × 0.010282] + [*ALG3* expression level × 0.005389] + [*PYGL* expression level × 0.004299] + [*DNMT1* expression level × (−0.014064)] + [*POLE2* expression level × (−0.077976)] + [*PDE6G* expression level × (− 0.081091)] + [*CYP2D6* expression level × (−0.160250)].

In the functional enrichment analysis of these 12 hub MRGs, the most significantly enriched term in the aspect of BP, CC, and MF was “hypoxanthine salvage,” “cytosol,” and “pyridoxal phosphate binding,” respectively (Figure 2F), whereas the significantly enriched KEGG pathways were “metabolic pathways” and “purine metabolism” (Figure 2G).

### 3.2 Evaluation of the MRGPS predicting ability in different data sets

**FIGURE 3:**
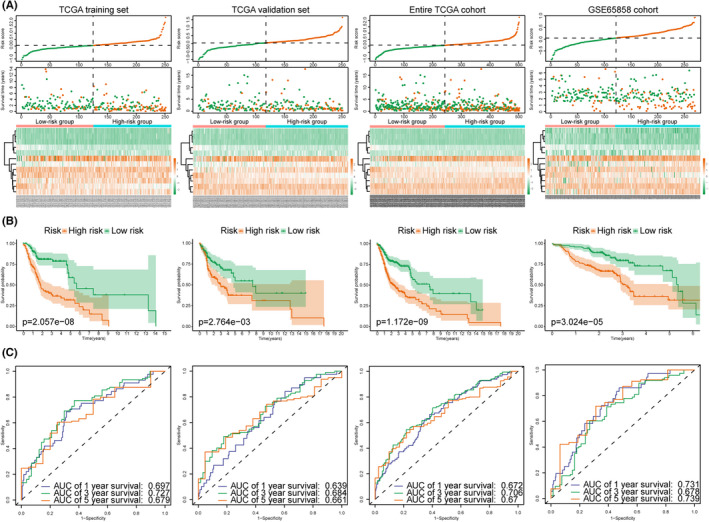
MRGPS accurately predicts the survival of HNSCC patients. Distribution of risk scores, survival status, as well as expression levels of 12 hub MRGs for HNSCC patients in low‐ and high‐risk groups (A), KM survival (B), and time‐dependent ROC curve (C) analyses of the TCGA training set, TCGA validation set, entire TCGA cohort, and GSE65858 cohort. HNSCC, head and neck squamous cell carcinoma; MRGPS, metabolism‐related gene‐based prognostic signature; MRGs, metabolism‐related genes; ROC, receiver‐operating characteristic; TCGA, The Cancer Genome Atlas

Based on the established MRGPS, we calculated the risk score for each HNSCC patient and classified all HNSCC patients into the low‐ or high‐risk group in accordance with the median risk score (0.049) (Figure 3A). According to our results, the low‐risk group presented favorable survival in comparison with the high‐risk group in multiple data sets (Figure 3B, *p* < 0.05). Furthermore, the time‐dependent ROC curves were plotted to evaluate the predicted performance of MRGPS (Figure 3C). It was calculated that the area under the curve values for 1‐, 3‐, and 5‐year survival were 0.697, 0.727, and 0.679, separately, in the TCGA training set, indicating that MRGPS performed well in monitoring survival. In the meanwhile, MRGPS was highly accurate in predicting survival of TCGA validation set, entire TCGA cohort, and GSE65858 cohort.

### 3.3 Relationships of MRGPS with patient prognosis and clinicopathologic variables

**FIGURE 4:**
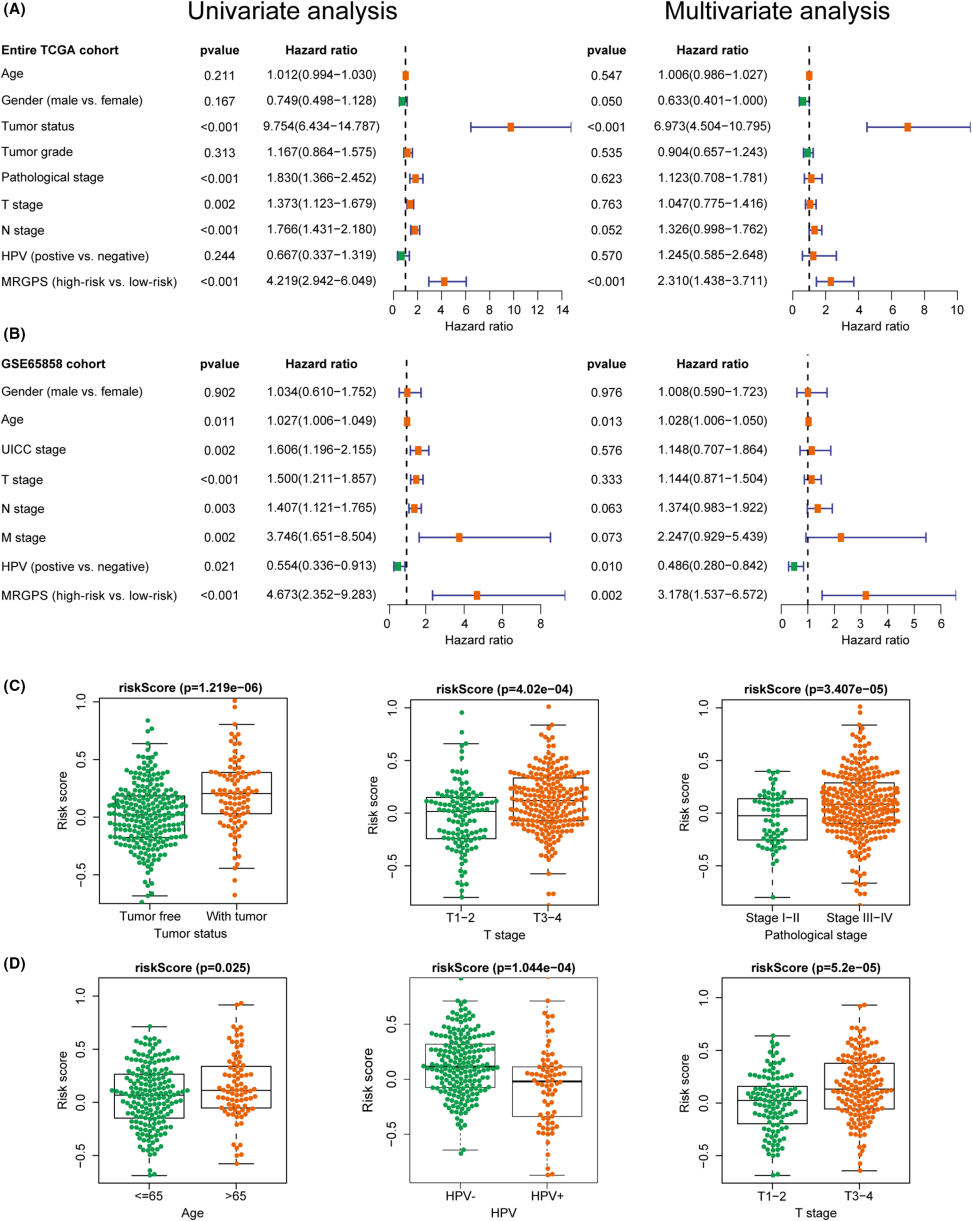
MRGPS is an independent prognostic indicator for HNSCC patients. Forest plot for univariate and multivariate Cox regression analyses of MRGPS and multiple clinicopathological variables in the entire TCGA cohort (A) and GSE65858 cohort (B). The correlations between the MRGPS and multiple clinicopathological variables in the entire TCGA cohort (C) and GSE65858 cohort (D). HNSCC, head and neck squamous cell carcinoma; MRGPS, metabolism‐related gene‐based prognostic signature; TCGA, The Cancer Genome Atlas

To explore the association of MRGPS and clinicopathologic variables with OS, we carried out Cox proportional hazards regression analysis on the entire TCGA cohort and the GSE65858 cohort. It was illustrated from Figure 4A,B that MRGPS was obviously related to OS in both univariate and multivariate analyses (*p* < 0.001), which showed that MRGPS might play the role of a factor in independently predicting the prognosis in the entire TCGA cohort along with GSE65858 cohort. Noteworthily, HPV status was also significantly correlated with OS in the GSE65858 cohort (Figure 4B). In addition, we explored the potential associations of MRGPS with multiple clinicopathologic variables. The risk score markedly increased in cases at advanced tumor status, T grade, and pathological stage in the entire TCGA cohort (*p* < 0.05, Figure 4C). Besides, the elderly cases, HPV‐negative cases, and those at advanced T stage in the GSE65858 cohort generally had high‐risk scores (*p* < 0.05, Figure 4D). According to the above results, MRGPS showed a significant correlation with several clinicopathologic variables.

Furthermore, we constructed a prognostic nomogram based on MRGPS and multiple clinicopathological variables, with the purpose of quantitatively estimating the survival probability among individual cases (Figure S2A). Typically, the calibration curves of our constructed prognostic nomogram were well consistent between the predicted and measured 1‐, 3‐, and 5‐year survivals in the entire TCGA cohort (Figure S2B–D).

### 3.4 Functional enrichment analysis by GSEA

**FIGURE 5:**
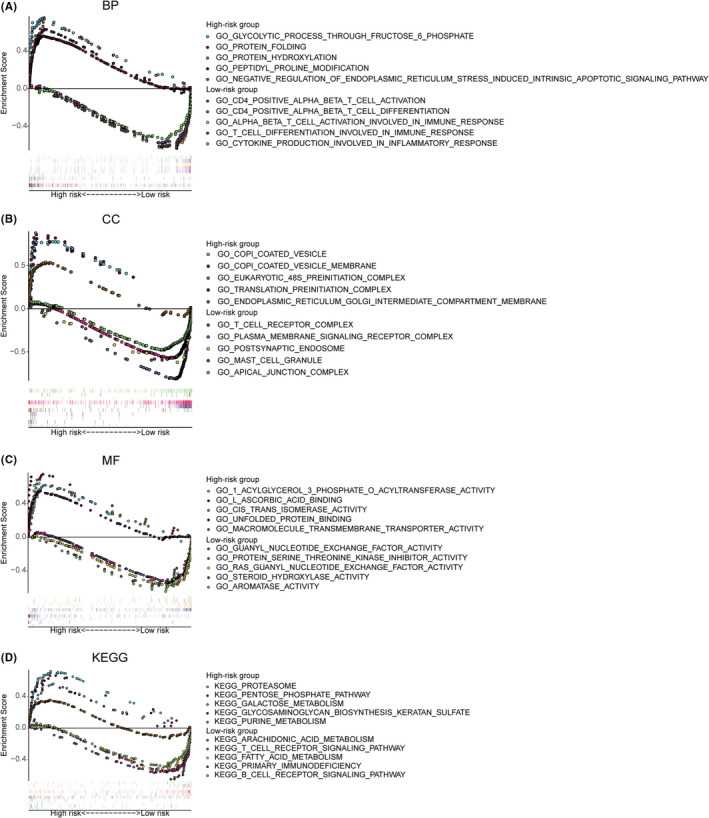
The enriched GO terms and KEGG pathways detected by GSEA. The representative GO terms of BP (A), CC (B), and MF (C) enriched in the high‐ and low‐risk group. (D) The representative KEGG pathways enriched in the high‐ and low‐risk groups. BP, biological process; CC, cellular component; GO, Gene Ontology; GSEA, gene set enrichment analysis; KEGG, Kyoto Encyclopedia of Genes and Genomes; MF, molecular function

To further study the underlying mechanism related to MRGPS, functional enrichment analysis was performed between high‐ and low‐risk groups via GSEA. The most significantly enriched BP in the high‐risk group was the “glycolytic process through fructose‐6‐phosphate” (Figure 5A). Intriguingly, in the low‐risk group, enriched BPs were closely correlated with immune response, such as “CD4 positive T cell activation,” “CD4 positive T cell differentiation,” and “T cell activation involved in immune response” (Figure 5A). In terms of CC (Figure 5B), the “T cell receptor complex” was the most obviously enriched component in the low‐risk group, further demonstrating that MRGPS was tightly related to immune response. In terms of MF, metabolism‐related terms were significantly enriched, including “acylglycerol 3 phosphate o acyltransferase activity,” “protein serine‐threonine kinase inhibitor activity,” and “steroid hydroxylase activity” (Figure 5C). Besides, GSEA‐enriched results of KEGG pathways revealed that several metabolism‐associated pathways were enriched in the high‐risk group, such as the “pentose phosphate pathway,” “galactose metabolism,” and “purine metabolism” (Figure 5D). In the low‐risk group, except for “arachidonic acid metabolism,” several immune‐related pathways were enriched, such as “T cell receptor and B cell receptor signaling pathways”, also indicating that MRGPS might be significantly associated with the immune response (Figure 5D).

### 3.5 Relationships of MRGPS with immune cells infiltration and immunotherapy

**FIGURE 6:**
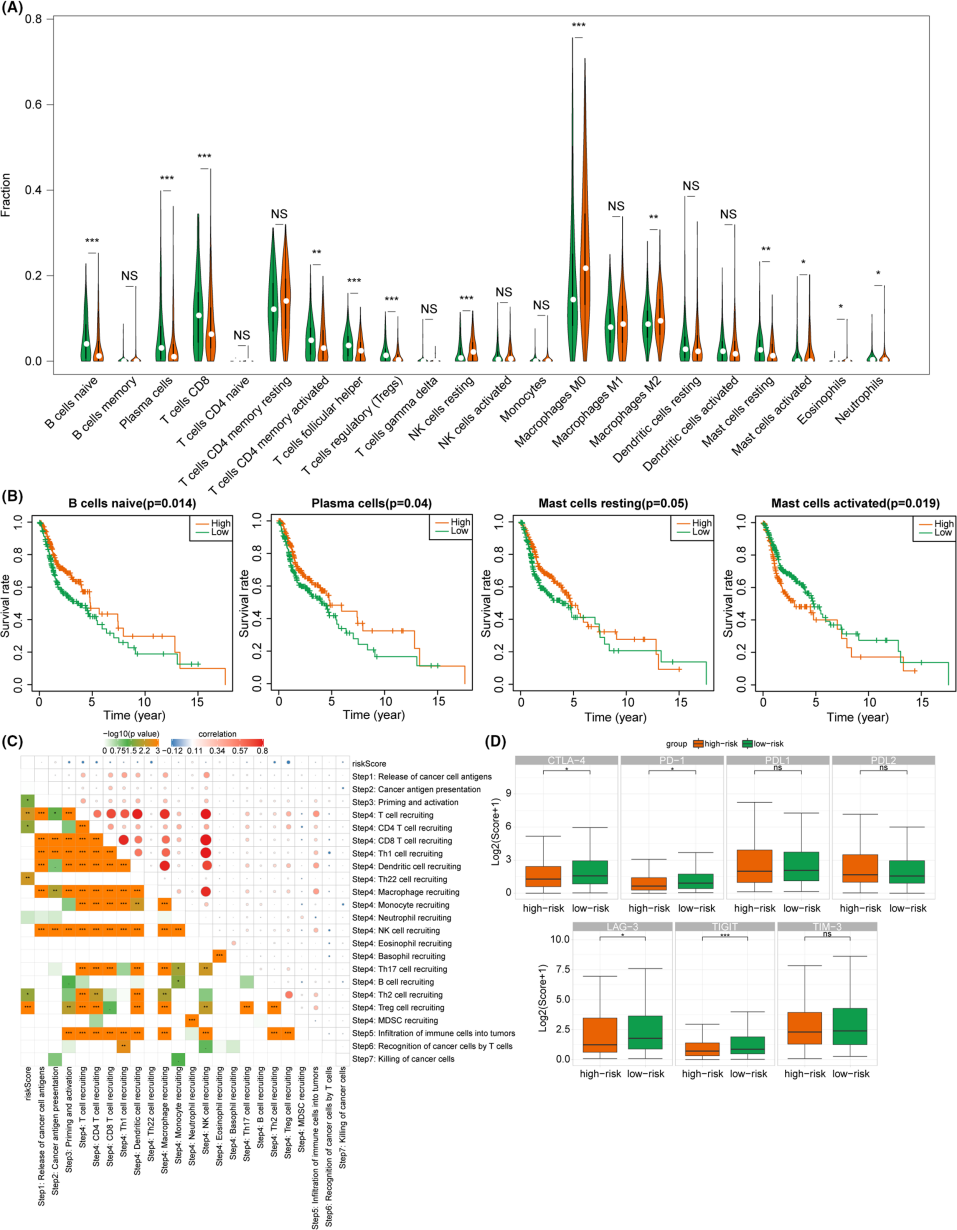
Relationship of MRGPS with tumor immune microenvironment. (A) Association between MRGPS and immune cell infiltration. The green and orange violins represented the low‐ and high‐risk groups, respectively. The white points inside the violin indicated median values. (B) Association of OS with naive B cells, plasma cells, resting mast cells, and activated mast cells. (C) Correlation matrix regarding MRGPS with the anticancer immune responses, where the red and blue dots represent positive and negative correlations, respectively. (D) The relationships between MRGPS and the expression of several major immune checkpoints. *, **, and *** represent *p* < 0.05, *p* < 0.01, and *p* < 0.001, respectively. MRGPS, metabolism‐related gene‐based prognostic signature; OS, overall survival

To further explore the relevance of MRGPS to immune response, the infiltration levels of 22 immune cell subtypes in all HNSCC samples were assessed by CIBERSORT. As shown in Figure 6A, the relative abundances of naive B cells, plasma cells, CD8 T cells, activated memory CD4 T cells, follicular helper T cells, and resting mast cells were significantly negatively correlated with a risk score, whereas those of resting NK cells, M0 and M2 macrophages were significantly positively correlated with risk score (*p* < 0.05). In addition, the prognostic value of these 22 immune cell subtypes was also analyzed. As a result, the infiltration levels of B cells, plasma cells, resting mast cells, as well as activated mast cells were markedly associated with survival rate (*p* < 0.05, Figure 6B). The increased infiltration levels of naive B cells, plasma cells, and resting mast cells predicted favorable OS, whereas that of activated mast cells predicted dismal OS. Besides, we explored the correlation between HPV status and immune cell infiltration, finding that HPV+ patients have more immune cell infiltration and better prognosis than HPV‐ patients (Figure S3).

The status of anticancer immune responses is an important part of the complex tumor immunophenotype underlying the tumor microenvironment. Therefore, we further analyzed the correlation between MRGPS and anticancer immune responses. It was illustrated from Figure 6C that MRGPS exhibited a negative correlation with T cell recruiting, CD4 T cell recruiting, Th22 cell recruiting, as well as priming and activation of immune (*p* < 0.05). Besides, ICI was a key component of immunotherapy. Therefore, we further investigated the associations between MRGPS and certain key immune checkpoints’ expression. As a result, the low‐risk group had higher levels of CTLA‐4, PD‐1, LAG‐3, and TIGIT compared with the high‐risk group (Figure 6D). Above all, MRGPS is correlated with immune cells infiltration and anticancer immune response to some extent.

### 3.6 Potential compounds targeting MRGPS

**FIGURE 7:**
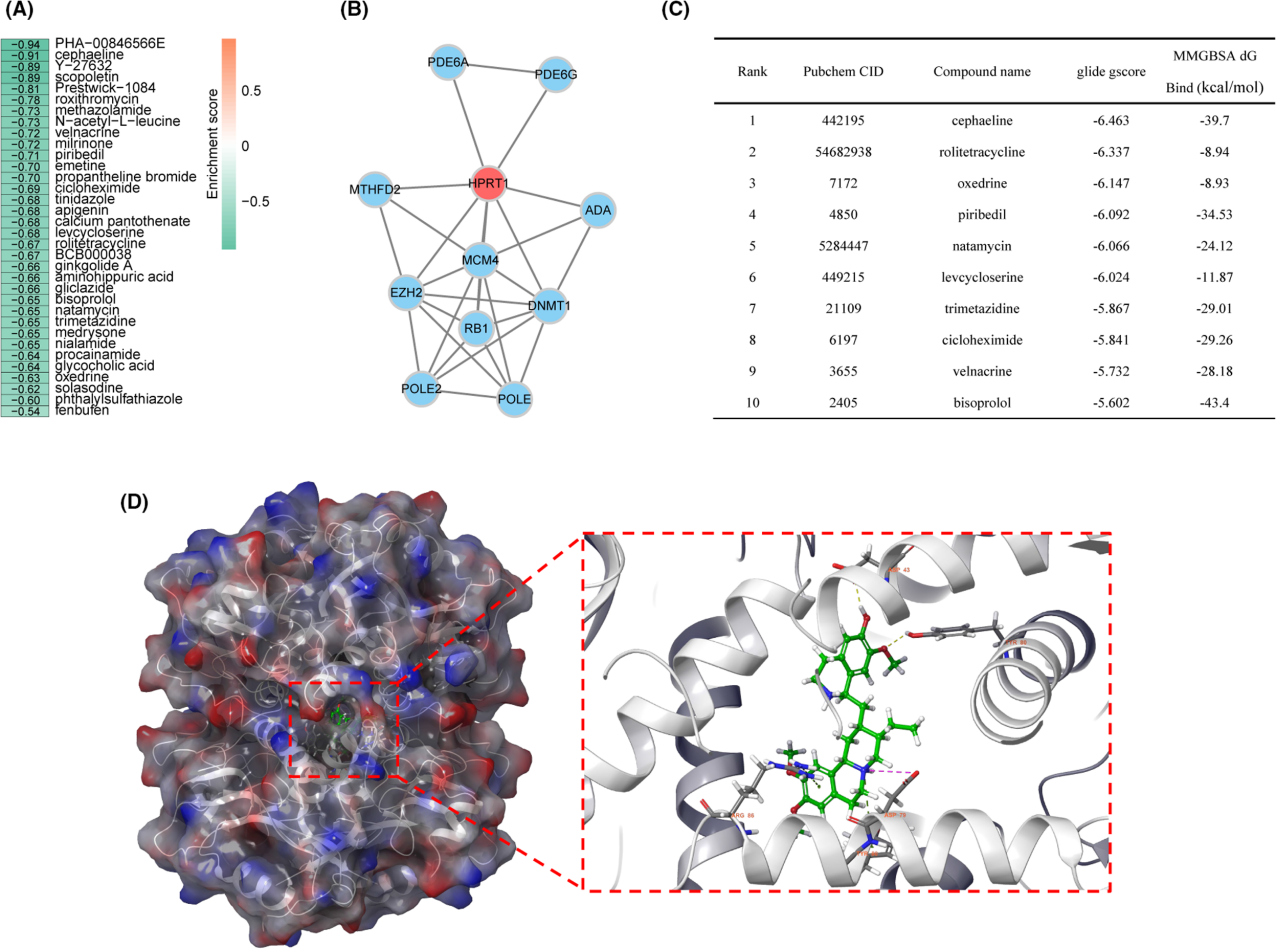
Screening of potential compounds. (A) Enrichment score of each potential compound from the Connectivity Map analysis. (B) The PPI network of MRGPS. (C) Top 10 compounds binding to key target HPRT1 based on molecular docking. (D) Structure and orthogonal view of the binding pocket between cephaeline and key target HPRT1. HPRT1, hypoxanthine phosphoribosyltransferase 1; MRGPS, metabolism‐related gene‐based prognostic signature; PPI, protein–protein interaction

In order to find out the potential compounds that target MRGPS, CMap analysis was conducted. According to the obtained results, a total of 34 compounds have the potential to reverse MRGPS (Figure 7A). Moreover, we constructed a PPI network using the genes associated with the signature and identified HPRT1 as a key target (Figure 7B and Table S3). For this key target, 34 candidate compounds were adopted for molecular docking with it. As shown in Figure 7C, we found the top 10 compounds among which cephaeline ranked first. The interaction diagram of cephaeline at the active site of HRPT1 showed hydrogen bonds’ formation with the key residues ASP‐43 and TYR‐80 (Figure 7D). Meanwhile, pi–pi stacking interactions with ARG‐86 and TRY‐80 also made contributions to stabilizing the ligand at the active sites. Apart from that, the relative binding free energy of cephaeline to the key target HPRT1 was also the lowest, indicating that their binding was stable. Therefore, we believe that cephaeline may be a potential therapeutic compound for HNSCC, which is worthy of further study.

## 4 DISCUSSION

Extensive studies have shown that cancer cells have abnormal metabolic behaviors such as the Warburg effect.^22^ Metabolic reprogramming is a vital hallmark in the development of cancer, which can facilitate cancer cell proliferation and invasion.^23^ Enhanced glycolysis is one of the characteristic features of HNSCC, and thus [18F]‐fluorodeoxyglucose‐positron emission tomography (FDG‐PET) that can reflect the glucose uptake is often used for clinical diagnosis and evaluation of HNSCC patients.^24^ Besides, frequent mutation of *P53* was found in HNSCC, which regulates the glycolysis pathway by inhibiting glucose intake, glycolysis flow, pentose phosphate pathway, and other pathways.^3^ Given the above, targeting energetic metabolism as an anticancer therapy is promising for HNSCC treatment.

In the present study, the expression of MRGs, together with their prognostic significance in HNSCC cases, was analyzed based on transcriptomic data. As a result, 12 MRGs were identified as hub MRGs and adopted for constructing MRGPS. Therefore, *P4HA1* was upregulated and significantly relevant to the clinical features of HNSCC.^25^ Besides, *ALG3* may play a key role in the oncogene expression in HNSCC, and its combined overexpression with *PPFIA1* is significantly associated with poor survival outcomes.^26^ Some genes, such as *CYP2D6*,^27^ *POLE2*,^28^ *DNMT1*,^29^, ^30^ *MTHFD2*,^31^, ^32^ and *PYGL*,^33^ have also been suggested to present significant associations with cancer prognostic outcomes. Although the other five genes have not been explored in HNSCC, their biological roles in HNSCC need to be further explored. Based on the median risk score (0.049), we classified HNSCC cases into the low‐ or high‐risk group. The obtained findings demonstrated that low‐risk patients had superior survival to high‐risk patients. Time‐dependent ROC curves revealed that MRGPS had a high potential for predicting survival. Besides, univariate or multivariate analysis indicated that MRGPS was an independent prognostic indicator.

Among the pathogenic factors of HNSCC, HPV infection is another crucial risk factor in addition to smoking and alcohol consumption. It is reported that HPV+ HNSCC patients tend to be slightly younger, male, and primarily non‐smokers.^34^ In addition, HPV+ HNSCC patients were often regarded as closely associated with improved prognosis than HPV− patients.^35^ Consistent with these reports, in the present study, HPV status was also significantly correlated with OS in the GSE65858 cohort in both univariate and multivariate analyses. Besides, HPV‐negative cases generally had higher risk scores than HPV‐positive cases. Therefore, HPV status was also included in the nomogram, which helps to predict patients’ survival better.

GSEA is a powerful tool for exploring the potential molecular mechanisms between different samples. By performing GSEA analysis, some metabolism‐associated pathways, such as “pentose phosphate pathway” and “purine metabolism,” were found to be enriched in the high‐risk group, whereas “arachidonic acid metabolism” was enriched in the low‐risk group. Notably, purine metabolism has been suggested to be associated with cancer progression, and purine nucleotides play crucial roles in providing cellular energy and intracellular signaling.^36^ Additionally, regulating the metabolism of arachidonic acid helps to regulate the occurrence of active inflammatory mediators. Noteworthily, low‐risk patients were associated with some immune pathways, which suggested that MRGPS might have an important connection with immune response. In recent years, numerous landmark studies have found that changes in metabolism may exert a vital part in immune regulation.^37^, ^38^ The abnormal metabolism of cancer cells can profoundly influence the tumor microenvironment that is commonly acidic, hypoxic, and depleted of essential nutrients necessary for immune cells.^39^ Furthermore, aerobic glycolysis within cancer cells assists in shaping the immune system through upregulating cytokine transcription while suppressing monocytes’ differentiation to dendritic cells.^40^ In addition, for immune cells themselves, metabolism is also a crucial determinant of viability and functions. As a result, metabolism is closely related to immunity, and the establishment of a metabolism‐related prognostic signature may contribute to predicting the state of the immune response.

To better explore the association of the MRGPS with immunity, this study compared TIME in the high‐risk group with that in the low‐risk group. As a result, the infiltration levels of a majority of immune cell subtypes, such as naive B cells, CD8 T cells, and activated memory CD4 T cells, enhanced in the low‐risk group in comparison with those in the high‐risk group. In further analyses, it could be discovered that the higher infiltration levels of naive B cells, plasma cells, and resting mast cells were associated with better OS, whereas the increased infiltration level of activated mass cells predicted dismal OS. These results were consistent with previous reports, pointing out that the metabolically active tumor cells created a detrimental microenvironment for immune cells.^41^ The anticancer immune response is another vital part of the complex tumor immunophenotype underlying the TIME. The abnormal metabolism possibly results in a diverse prognosis through altering immune cell state within TIME. This conforms to the report that targeted metabolism can be conducive to regulating the antitumor immune response.

Some strengths should be noted in the present work. First of all, this is the first constructed signature based on metabolic reprogramming that can reflect the prognosis and TIME of HNSCC patients. Second, multiple data sets were adopted for validating and evaluating the predictive performance of our constructed signature. Third, we identified the potential compound that targeted our constructed signature. Nevertheless, further prospective cohort studies should be conducted to assess the clinical value of this prognostic signature. Meanwhile, the potential compound needs to be further explored in the future.

## 5 CONCLUSIONS

In summary, we conducted a comprehensive analysis of the expression patterns of MRGs in HNSCC patients and established an MRGs‐based signature that has the potential to predict the clinical outcome and immune microenvironment. As an effective tool, this signature may help to search for potential combination immunotherapy compounds and provide a promising therapeutic strategy for treating HNSCC patients.

## CONFLICT OF INTEREST

The authors declare that they have no competing interests.

## AUTHOR CONTRIBUTIONS

Weijie Qiang, Yifei Dai, and Xiaobo Sun conceived the study; Weijie Qiang and Yifei Dai designed and performed the experiments; Weijie Qiang wrote the manuscript; Yifei Dai and Xiaoyan Xing edited the manuscript; all authors read and gave final approval to submit the manuscript.

## ETHICAL APPROVAL STATEMENT

All analyses were based on previously published studies, thus no ethical approval and patient consent are required.

## Supporting information


Figure S1
Figure S2
Figure S3
Table S1
Table S2
Table S3

## Data Availability

The data used to support the findings of this study are available from the TCGA (https://portal.gdc.cancer.gov/) and GEO data sets (https://www.ncbi.nlm.nih.gov/gds/).
