# Supplementary material for: Identification of a metabolic reprogramming‐related signature associated with prognosis and immune microenvironment of head and neck squamous cell carcinoma by in silico analysis
Source: Cancer Med. 2022 Mar 18;11(16):3168–81. doi: 10.1002/cam4.4670 (PMC9385599; doi:10.1002/cam4.4670)
Supplement: Supplementary file 1 — Figure S1 Figure S2 Figure S3 Table S1 Table S2 Table S3 [file CAM4-11-3168-s001.docx]

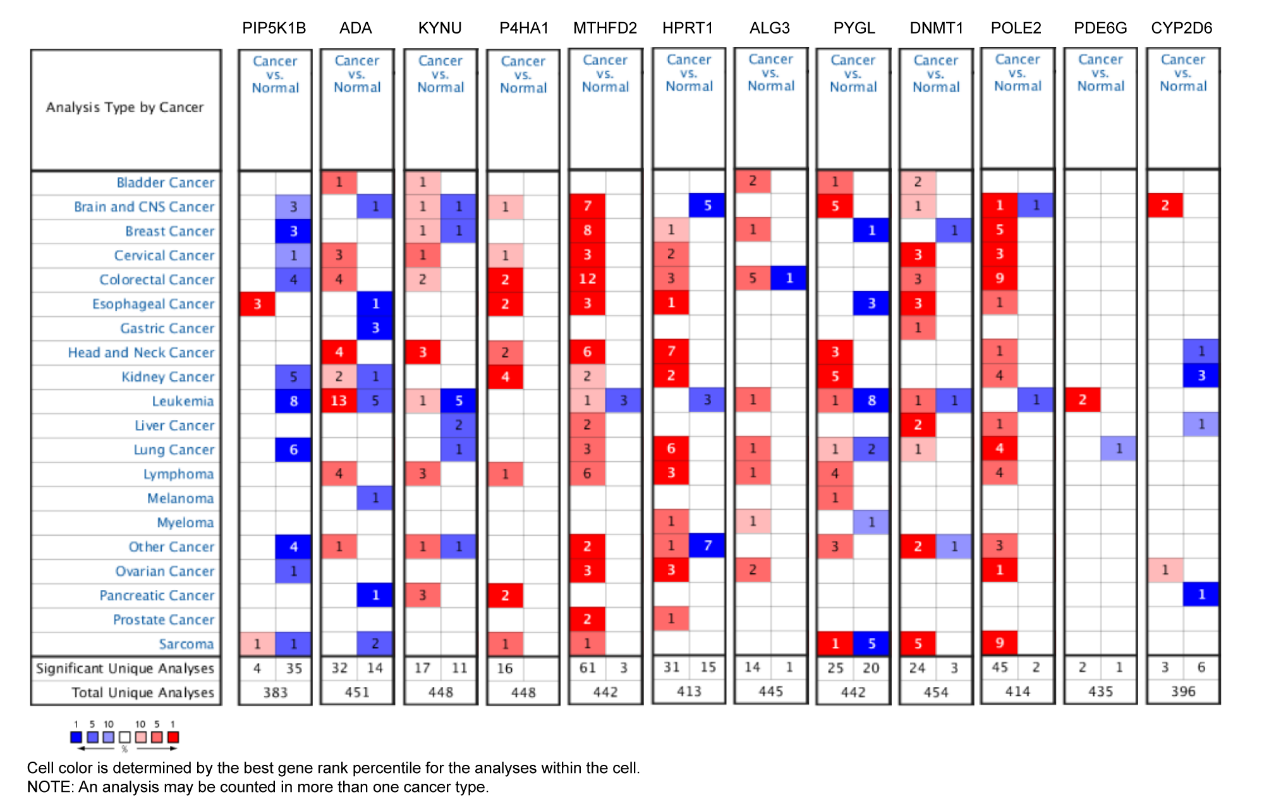


**Fig. S1** Gene expression levels of 12 MRGs contained in MRGPS (based on the Oncomine database).


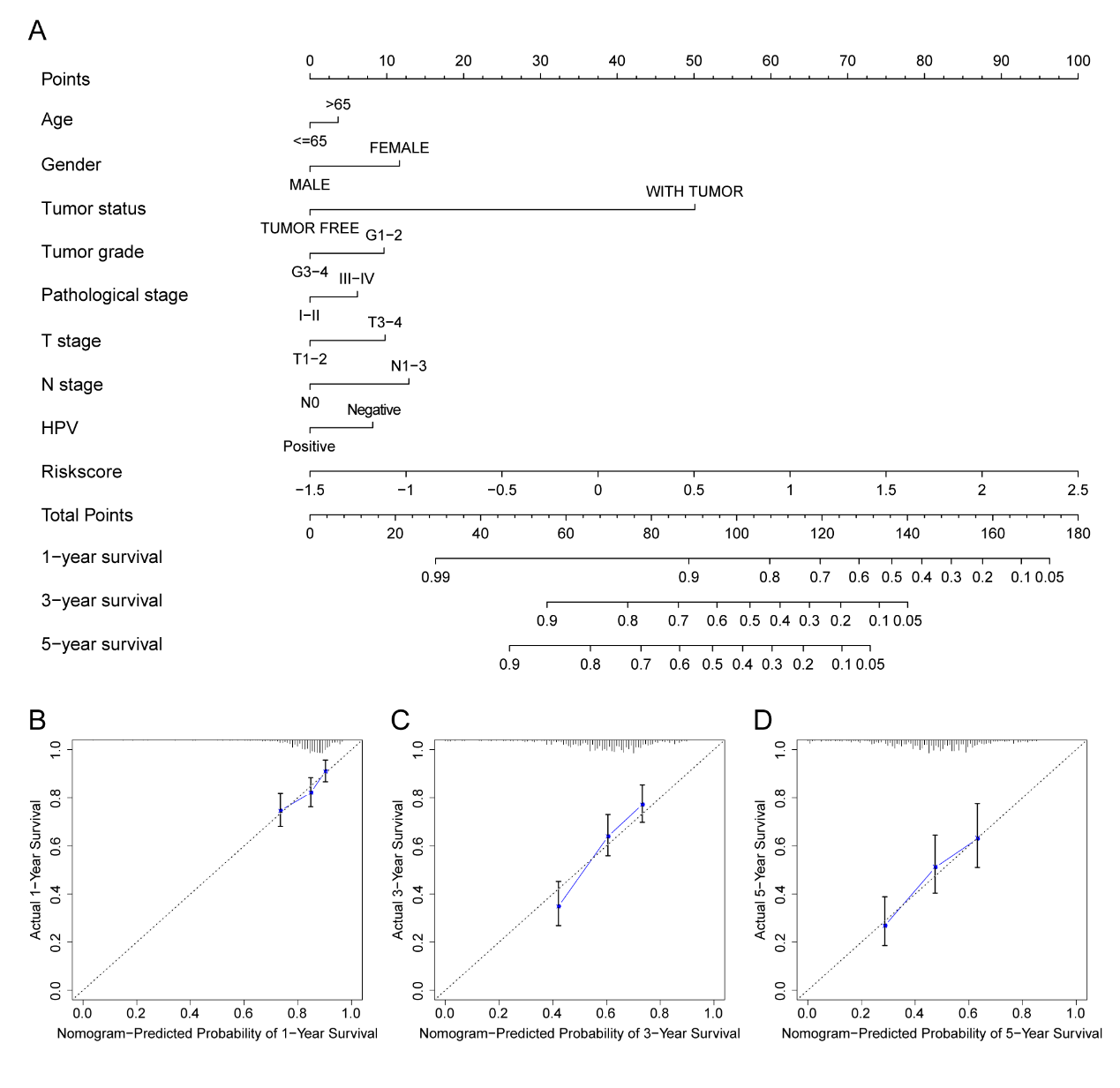


**Fig. S2** The prognostic nomogram of HNSCC patients based on MRGPS and other clinicopathological factors. Nomogram for predicting 1‐, 3‐, and 5‐year OS in entire TCGA cohort (A). Calibration curves of nomogram on consistency between predicted and observed 1‐, 3‐, and 5‐year survival in entire TCGA cohort (B-D). Dashed line at 45° implicated a perfect prediction, and the actual performances of our nomogram were shown in blue lines.


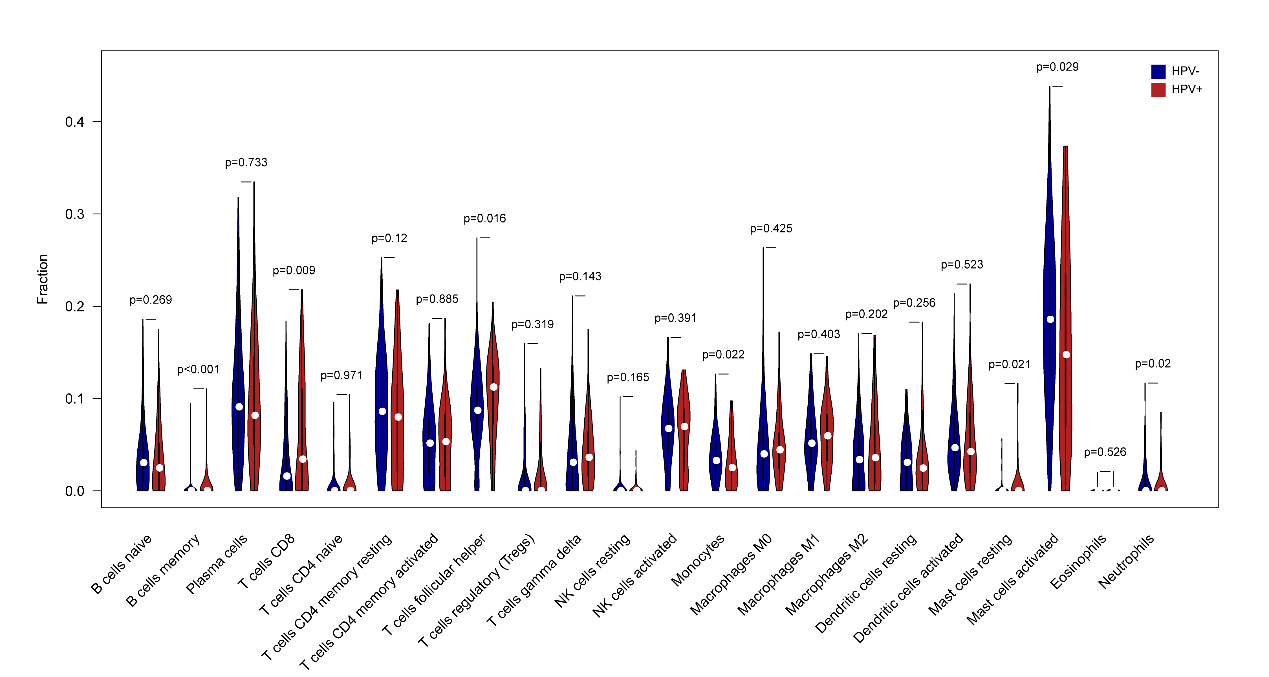


**Fig. S3** The association between HPV status and immune cell infiltration. The blue and red violin represented the HPV- and HPV+ patients, respectively. The white points inside the violin implicated median values.

**Tab. S1** Patient demographics and clinical characteristics of the included datasets.

| Variables | | Group | Entire TCGA cohort (n = 502) | TCGA training set (n = 251) | TCGA validation set (n = 251) | GSE65858 cohort (n = 270) |
| --- | --- | --- | --- | --- | --- | --- |
| Survival time(days) | | | 840±38.82* | 878±60.98 | 798±48.06 | 883±27.49 |
| Vital status | | Alive | 306 (61.0%) | 157 (62.5%) | 149 (59.4%) | 176 (65.2%) |
|  | | Dead | 196 (39.0%) | 94 (37.5%) | 102 (40.6%) | 94 (34.8%) |
| Gender | | Female | 134 (26.7%) | 68 (27.1%) | 66 (26.3%) | 47 (17.4%) |
|  | | Male | 368 (73.3%) | 183 (72.9%) | 185 (73.7%) | 223 (82.6%) |
| Age | | <=65 | 325 (64.7%) | 155 (61.8%) | 170 (67.7%) | 184 (68.1%) |
|  | | >65 | 176 (35.1%) | 96 (38.2%) | 80 (31.9%) | 86 (31.9%) |
|  | | NA | 1 (0.2%) | 0 | 1 (0.4%) | 0 |
| Cancer status | | Tumor free | 316 (62.9%) | 163 (64.9%) | 153 (61.0%) |  |
|  | | With tumor | 138 (27.5%) | 65 (25.9%) | 73 (29.1%) |  |
|  | | NA | 48 (9.6%) | 23 (9.2%) | 25 (10.0%) |  |
| Tumor grade | | G1 | 62 (12.4%) | 28 (11.2%) | 34 (13.5%) |  |
|  | | G2 | 300 (59.8%) | 148 (59.0%) | 152 (60.6%) |  |
|  | G3 | 119 (23.7%) | 67 (26.7%) | 52 (20.7%) |  |  |
|  | | G4 | 2 (0.4%) | 0 | 2 (0.8%) |  |
|  | | GX | 19 (3.8%) | 8 (3.2%) | 11 (4.4%) |  |
| Pathological stage | | Stage I | 25 (5.0%) | 11 (4.4%) | 14 (5.6%) |  |
|  | | Stage II | 70 (13.9%) | 39 (15.5%) | 31 (12.4%) |  |
|  | | Stage III | 78 (15.5%) | 41 (16.3%) | 37 (14.7%) |  |
|  | | Stage IV | 261 (52%) | 123 (49.0%) | 138 (55.0%) |  |
|  | | NA | 68 (13.5%) | 37 (14.7%) | 31 (12.4%) |  |
| T stage | | T1 | 45 (9.0%) | 21 (8.4%) | 24 (9.6%) | 35 (13%) |
|  | | T2 | 133 (26.5%) | 66 (26.3%) | 67 (26.7%) | 80 (29.6%) |
|  | | T3 | 96 (19.1%) | 46 (18.3%) | 50 (19.9%) | 58 (21.5%) |
|  | | T4 | 172 (34.3%) | 86 (34.3%) | 86 (34.3%) | 97 (35.9%) |
|  | | TX | 56 (11.2%) | 32 (12.7%) | 24 (9.6%) |  |
| M stage | | M0 | 188 (37.5%) | 83 (33.1%) | 105 (41.8%) | 263 (97.4%) |
|  | | M1 | 1 (0.2%) | 0 | 1 (0.4%) | 7 (2.6%) |
|  | | MX | 313 (62.4%) | 168 (66.9%) | 145 (57.8%) |  |
| N stage | | N0 | 171 (34.1%) | 91 (36.3%) | 80 (31.9%) | 94 (34.8%) |
|  | | N1 | 65 (12.9%) | 36 (14.3%) | 29 (11.6%) | 32 (11.9%) |
|  | | N2 | 166 (33.1%) | 72 (28.7%) | 94 (37.5%) | 132 (48.9%) |
|  | | N3 | 7 (1.4%) | 5 (2.0%) | 2 (0.8%) | 12 (4.4%) |
|  | | NX | 93 (18.5%) | 47 (18.7%) | 46 (18.3%) |  |
| UICC stage | | I |  |  |  | 18 (6.7%) |
|  | | II |  |  |  | 37 (13.7%) |
|  | | III |  |  |  | 37 (13.7%) |
|  | | IV |  |  |  | 178 (65.9%) |
| HPV | | Negative | 413 (82.3%) | 207 (82.5%) | 206 (82.1%) | 196 (72.6%) |
|  | | Positive | 70 (13.9%) | 31 (12.4%) | 39 (15.5%) | 73 (27.0%) |
|  | | NA | 19 (3.8%) | 13 (5.2%) | 6 (2.4%) | 1 (0.4%) |

NA: Not Available; TX: unknown T stage; MX: unknown M stage; GX: unknown pathological grade; NX: unknown N stage. * The data are presented as mean ± SE.

**Tab. S2** 38 DEMRGs were significantly relevant to the OS of HNSCC patients based on univariate Cox proportional hazard regression analysis (*P* <0.05). The 12 genes that constructed MRGPS were shown in bold.

| Characteristics | Hazard_Ratio | CI95 | P_value |
| --- | --- | --- | --- |
| *ADH7* | 1.01 | 1 - 1.01 | 0.034 |
| *GLA* | 1.03 | 1 - 1.05 | 0.02284 |
| *ACAT1* | 1.09 | 1.04 - 1.14 | 0.00081 |
| *SQLE* | 1.01 | 1 - 1.02 | 0.03459 |
| *DHCR7* | 1 | 1 - 1.01 | 0.00361 |
| *CYP27B1* | 1.04 | 1 - 1.09 | 0.04647 |
| *COX6B2* | 0.87 | 0.77 - 0.98 | 0.01923 |
| ***ADA*** | 1.03 | 1.02 - 1.05 | 0.00006 |
| ***POLE2*** | 0.9 | 0.81 - 0.99 | 0.03812 |
| *POLD1* | 0.96 | 0.93 - 0.99 | 0.02418 |
| *POLE* | 0.86 | 0.76 - 0.96 | 0.0068 |
| ***PDE6G*** | 0.58 | 0.36 - 0.93 | 0.02298 |
| ***HPRT1*** | 1.03 | 1.02 - 1.04 | 0 |
| *TXNRD1* | 1.01 | 1 - 1.01 | 0.02153 |
| *ASNS* | 1.03 | 1.01 - 1.05 | 0.00125 |
| *GATM* | 0.91 | 0.85 - 0.99 | 0.02143 |
| *CHDH* | 0.59 | 0.41 - 0.85 | 0.00434 |
| ***DNMT1*** | 0.96 | 0.93 - 0.98 | 0.00205 |
| *SMS* | 1.01 | 1 - 1.01 | 0.00047 |
| *PLOD2* | 1.02 | 1 - 1.04 | 0.01459 |
| ***P4HA1*** | 1.02 | 1.01 - 1.03 | 0.00037 |
| ***KYNU*** | 1.06 | 1.01 - 1.12 | 0.02653 |
| *SCLY* | 0.17 | 0.04 - 0.78 | 0.0228 |
| *AMY2B* | 0.39 | 0.17 - 0.88 | 0.02411 |
| ***PYGL*** | 1.01 | 1 - 1.01 | 0.00364 |
| ***ALG3*** | 1.01 | 1 - 1.02 | 0.00565 |
| *MAN1C1* | 0.68 | 0.47 - 0.98 | 0.03835 |
| *FUCA2* | 1.02 | 1 - 1.04 | 0.04536 |
| *EXT2* | 1.01 | 1 - 1.01 | 0.04111 |
| *HS3ST1* | 1.12 | 1.03 - 1.21 | 0.00802 |
| ***PIP5K1B*** | 1.11 | 1.03 - 1.2 | 0.00733 |
| *MINPP1* | 1.08 | 1.01 - 1.15 | 0.01579 |
| *PTDSS1* | 1.02 | 1 - 1.03 | 0.01805 |
| *PLA2G2D* | 0.86 | 0.78 - 0.96 | 0.00598 |
| *ACACB* | 0.68 | 0.48 - 0.97 | 0.03099 |
| ***MTHFD2*** | 1.04 | 1.02 - 1.05 | 0.00001 |
| *ACSM3* | 0.32 | 0.11 - 0.96 | 0.04191 |
| ***CYP2D6*** | 0.24 | 0.1 - 0.56 | 0.00112 |

**Tab. S3** The topological analysis results of the PPI network of MRGPS.

| Name | Degree Centrality | Betweenness Centrality | Closeness Centrality |
| --- | --- | --- | --- |
| **HPRT1** | **8** | **0.396296** | **0.833333** |
| MCM4 | 8 | 0.118519 | 0.833333 |
| EZH2 | 7 | 0.07037 | 0.769231 |
| DNMT1 | 7 | 0.07037 | 0.769231 |
| RB1 | 6 | 0.033333 | 0.714286 |
| POLE | 5 | 0 | 0.588235 |
| POLE2 | 5 | 0 | 0.588235 |
| MTHFD2 | 3 | 0 | 0.588235 |
| ADA | 3 | 0 | 0.588235 |
| PDE6G | 2 | 0 | 0.5 |
